# Genetically Engineered Islets and Alternative Sources of Insulin-Producing Cells for Treating Autoimmune Diabetes: Quo Vadis?

**DOI:** 10.1155/2012/296485

**Published:** 2012-05-29

**Authors:** Feng-Cheng Chou, Shing-Hwa Huang, Huey-Kang Sytwu

**Affiliations:** ^1^Department and Graduate Institute of Microbiology and Immunology, National Defense Medical Center, Neihu, Taipei 114, Taiwan; ^2^Department of General Surgery, Tri-Service General Hospital, Taipei 114, Taiwan

## Abstract

Islet transplantation is a promising therapy for patients with type 1 diabetes that can provide moment-to-moment metabolic control of glucose and allow them to achieve insulin independence. However, two major problems need to be overcome: (1) detrimental immune responses, including inflammation induced by the islet isolation/transplantation procedure, recurrence autoimmunity, and allorejection, can cause graft loss and (2) inadequate numbers of organ donors. Several gene therapy approaches and pharmaceutical treatments have been demonstrated to prolong the survival of pancreatic islet grafts in animal models; however, the clinical applications need to be investigated further. In addition, for an alternative source of pancreatic **β**-cell replacement therapy, the *ex vivo* generation of insulin-secreting cells from diverse origins of stem/progenitor cells has become an attractive option in regenerative medicine. This paper focuses on the genetic manipulation of islets during transplantation therapy and summarizes current strategies to obtain functional insulin-secreting cells from stem/progenitor cells.

## 1. Introduction

Type 1 diabetes (T1D) is an autoimmune disease characterized by the progressive destruction of insulin-producing cells in the pancreatic islets by autoreactive T cells, which eventually leads to hyperglycemia. The disease accounts for about 10% of all cases of diabetes, occurs most commonly in people of European descent, and affects two million people in Europe and North America [1]. There is a marked geographic variation in the incidence, probably because the different populations vary in genetic susceptibility/resistance factors or in exposure to environmental triggers. For instance, a child in Finland (Northern Europe) is about 80 times more likely to acquire the disease than a child in China (Eastern Asia) [2]. The current global increase in incidence of 3% per year is well established [3, 4], and this rapid rise strongly suggests that environmental factors should be acting on susceptibility genes and contributing to the evolving epidemiology of T1D.

In patients with T1D, daily delivery of insulin by injection or a pump is crucial for metabolic control. However, this exogenous insulin delivery cannot achieve physiological control of blood glucose concentrations and also has the risk of causing hypoglycemic episodes. Moreover, a significant proportion of patients suffers chronic and degenerative complications, such as nephropathy, retinopathy, and vascular and heart disease [5, 6]. The appropriate treatment to achieve insulin independence for T1D is replacement of the *β*-cell mass, currently being accomplished through whole pancreas transplantation and islet transplantation. Transplantation of the whole pancreas is a standard treatment for diabetic patients, which can achieve insulin independence with a single donor [7]. Pancreatic islet transplantation is a safer and less invasive method than whole-organ transplant therapy, which causes thrombosis, pancreatitis, and peritonitis. However, the major drawback of islet transplantation compared with pancreas transplantation is the greater requirement for donors and the lower 5-year insulin independence rate [8].

Transplantation therapy should provide a better quality of life than current therapies and should help avoid complications. Unfortunately, immune-mediated destruction and inadequate numbers of donor organs for transplantation are the major obstacles to achieving insulin independence and long-term survival of grafts in this therapy. To circumvent those problems, genetically modifying islets to enhance their resistance to immune attack and explorations of alternative sources of insulin-secreting cells are being investigated intensively. In this paper, we will summarize the current knowledge regarding immunomodulatory therapy in islet transplantation and examine alternative sources of insulin-secreting cells for cell replacement therapy.

## 2. Mechanisms Involved in Islet Graft Rejection: Nonspecific Inflammation and the Contribution of T-Helper-Cell Subsets

The process of islet isolation also triggers a cascade of stressful events in the cells involving the induction of apoptosis or necrosis and production of proinflammatory molecules that negatively influence islet viability and function. Transplantation procedures such as collagenase-based islet isolation trigger proinflammatory cytokine and chemokine production by the islets. Proinflammatory cytokines such as interleukin- (IL-) 1*β* and tumor necrosis factor- (TNF-) *α* produced by islet-resident macrophages are toxic to islets and can induce the local production of reactive oxygen species (ROS) [9, 10]. By contrast, chemokine receptors such as chemokine (C-C motif) receptor (CCR)2, CCR5, and C-X-C chemokine receptor (CXCR)3, and their ligands are crucial to generate acute islet allograft rejection [11]. Taken together, the inflammatory cytokines, chemokines, and ROS contribute to the first line of attack to the islets, which can cause apoptosis and loss of function.

The second barrier against successful transplantation is recipient alloimmunity and autoimmunity. Previous reports have demonstrated that Th1 cells, type 1 cytotoxic CD8^+^ T cells, and Th1-type cytokines such as interferon (IFN)-*γ* and IL-2 are commonly associated with graft rejection [12–15]. Th1 responses initiate allograft rejection by promoting cytotoxic T-cell activities and IFN-*γ*-mediated delayed-type hypersensitivity reactions, whereas Th2 responses cause allograft damage through the recruitment of eosinophils induced by IL-4 and IL-5. Moreover, cytokines produced by non-T-cell sources from the graft microenvironment, such as IL-7 produced by stromal cells and IL-15 produced by activated macrophages and endothelial cells, further support the idea of alloreactive T-cell proliferation [14, 16].

Recently, the role of subsets of Th cells in graft rejection has been reexamined after the identification and thorough characterization of Th17 cells. Several lines of evidence have demonstrated that Th17 cells have the capacity to cause rejection of cardiac allografts [17–19]. However, it is not clear whether Th1 and Th17 cells work synergistically or sequentially to cause graft rejection. In our laboratory, we have investigated the relative contribution of Th1 and Th17 cells in the autoimmune-mediated rejection of islets in a nonobese diabetic (NOD) mouse model. We have demonstrated that Th1 cells play a crucial role in the destruction of the islet graft, whereas Th17 cells constitute a much smaller population in the islet graft and might play only a minor destructive role in this model of autoimmune islet transplantation (Chou et al., manuscript in revision).

In summary, understanding the inflammatory factors that attack islets during the early phase and dissecting the role of the effector T-cell subsets in the rejection responses might contribute to developing target therapies to protect islets from inflammatory insults and to modulate T-cell responses.

## 3. Strategies to Protect Islets from Inflammatory Insults and T-Cell-Mediated Immunity

In autoimmune diabetes, pancreatic *β* cells suffer from inflammatory stress following T-cell-mediated destruction. Macrophages and/or dendritic cells in the islet microenvironment produce proinflammatory cytokines and free radicals, which induce *β*-cell damage. Activated T cells express death receptors and release cytotoxic molecules including granzyme B, perforin, or cytokines to further activate other immune cells and exacerbate *β*-cell death. Several preventive and therapeutic approaches have been demonstrated to protect *β* cells from immune attack, including the modulation of T-cell activity and inhibition of inflammatory responses in the islet microenvironment. To protect islets from immune attack, many gene targets that exhibit strong immunoregulatory effects and antiapoptotic effects have been introduced to the islets through different approaches: generation of transgenic mice using islet-specific promoters to carry genes of interest, delivery of genes into islets by viral vectors or transfection, and the administration of recombinant proteins and drugs.

## 4. Transgenic Overexpression of Regulatory Genes in Islets

Genetically manipulating islets by transgenic techniques was originally designed for study of the immunopathogenesis of autoimmune diabetes. This could help in dissecting the roles of different cytokines, death receptors, and major histocompatibility complex (MHC)/costimulation molecules in *β*-cell destruction (reviewed in [20]). Among the transgenic-mouse models that have been generated, some molecules display strong immunoregulatory functions and cytoprotective effects, which could be applied further in islet transplantation therapy (Table 1).

### 4.1. Cytokines and Cytokine Signaling

It is well established that proinflammatory cytokines and Th1-type cytokines are toxic to islets whereas IL-4 and transforming growth factor- (TGF-) *β* are postulated to be protective. Transgenic expression of IL-4 in *β* cells under the control of the insulin promoter in NOD mice suppresses insulitis and diabetes; however, islet expression of IL-4 is incapable of preventing islet rejection in diabetic recipients [21]. In other studies, expression of TGF-*β* driven by an insulin promoter [22] or a glucagon promoter [24] protected islets from autoimmune destruction in NOD mice. However, the *β*-cell-specific expression of TGF-*β* changes the pancreatic architecture [22], and this TGF-*β*-expressing pancreatic tissue fails to inhibit allograft rejection [23]. In other aspects, the inhibition of toxic cytokine signaling in islets represents an attractive strategy in designing therapies to prevent islet destruction. Islets with transgenic expression of suppressor of cytokine signaling 1 (SOCS1) show delayed allograft rejection but cannot circumvent destruction of the islets by the autoimmune destruction [26].

### 4.2. Negative Costimulation Engagement

T-cell activation occurs through two important signals: one is the T-cell receptor recognizing a specific peptide MHC complex and the other is a costimulatory signal. Upon the T-cell activation, the expression of negative costimulatory molecules is induced. The programmed death (PD)-1 and cytotoxic T-lymphocyte antigen (CTLA)-4 are two important negative costimulatory molecules expressed on T cells, which control their effector functions, tolerance, and autoimmunity [36]. We have demonstrated that transgenic expression of PD-L1 (ligand of PD-1) [27] or a membrane-bound, agonistic single-chain anti-CTLA-4 Fv antibody (anti-CTLA-4 scFv) [30] on islets in NOD mice reduces the severity of insulitis and suppresses the development of diabetes. In an islet transplantation study, transgenic anti-CTLA-4 scFv prolonged islet graft survival and reduced the Th1 cell counts in islet grafts after transplantation into spontaneous diabetic NOD mice. However, the expression of PD-L1 on islets could not prolong graft survival [27]. The role of PD-L1 in the regulation of T-cell tolerance to islets needs to be further investigated because the transgenic expression of PD-L1 on islets in mice with a B6 background induced T-cell-mediated spontaneous diabetes, and the islets from transgenic mice displayed accelerated rejection in an allogeneic transplantation model [29].

### 4.3. Anti-Inflammatory, Antiapoptotic, and Antioxidative Molecules

Inflammatory cytokines such as IL-1*β*, TNF-*α*, and IFN-*γ* sensitize *β* cells to Fas-dependent and/or other death receptor-mediated apoptosis [37] and induce ROS formation in *β* cells. Because islets produce very low levels of antioxidative enzymes and are very sensitive to oxidative stress [38], the reduction of ROS levels in islets is crucial for maintaining the function and viability of islets. Others and we have demonstrated that *β*-cell-specific expression of the antiapoptotic and anti-inflammatory proteins, thioredoxin (TRX) [32] or heme oxygenase-1 (HO-1) [33], prevented autoimmune diabetes in NOD mice. Moreover, the islets from HO-1 transgenic mice survived longer in diabetic recipients, indicating that control of the initial inflammatory responses can promote graft survival. The roles of ROS scavengers in islet transplantation have also been investigated in transgenic mouse models. Thus, several *β*-cell-specific transgenic mice with different antioxidant enzymes have been generated (reviewed in [39]). In general, islets with transgenic antioxidative genes (e.g., catalase, glutathione peroxidase, metallothionein, copper/zinc superoxide dismutase, and manganese superoxide dismutase) are resistant to oxidative stress induced by chemicals [40–42] or hypoxia [43]; however, they are still sensitive to proinflammatory cytokines induced cytotoxicity [41]. Among these transgenic mice, islets from metallothionein transgenic mice showed prolonged survival of islet grafts in an allogeneic transplantation model [43].

In summary, most *ex vivo* studies have shown that overexpression of antioxidative genes in islets protects them from oxidative injury; however, the *in vivo* function and survival of these genetically modified islets in diabetic recipients have not produced overt success.

## 5. Genetically Engineering Islets by Transfection or Transduction

The direct delivery of protective and therapeutic genes to islet grafts can overcome many problems; for example, the therapeutic agents cannot be targeted locally and might have effects on other organs or tissues, causing unexpected side effects. By using gene therapy, islets can be manipulated by any vector system *ex vivo* without exposing the recipient to the vectors. Moreover, graft-specific gene therapy can provide prolonged, safe, and locally controlled gene expression. In this regard, *ex vivo* manipulation of islets by gene transfer systems becomes an attractive approach to protect grafts from immune attack. However, the gene delivery systems applied should be considered carefully. In general, nonimmunogenic vectors that cannot activate the host's immune response are used for long-term gene expression [44].

Many strategies have been proven to improve the function of islet grafts and protect grafts from immune attack (Table 2). These approaches include blockade of co-stimulation signals by CTLA-4-Ig [45, 46]; downregulation of Th1 responses by overexpression of galectin-9 (Chou et al., manuscript in revision); overexpression of antiapoptotic and antioxidative molecules such as B-cell lymphoma (Bcl)-2 [47], TRX [48], and superoxide dismutases (SODs) [49]; blockade of inflammatory cytokine signaling by overexpression of IL-1 receptor antagonist protein [50]; overexpression of anti-inflammatory cytokines such as TGF-*β* [51], IL-10 [52], and IL-4 [53].

In summary, these strategies significantly reduce apoptosis in islet grafts and prolong graft survival in diabetic recipients. However, the application of these protective genes to transplantation therapy has not been successful. In general, therapeutic targets that have paracrine actions would exert more marked biological effects than membrane-bound or intracellular molecules. Moreover, the efficiency of gene delivery to islets and the expression levels of target proteins in the microenvironment of grafts are closely linked to the protective effect in the grafts. Therefore, better results might have been obtained by using a “cocktail” therapy, for example, combining antiapoptotic and anti-inflammatory genes, which could display synergistic protective effects.

## 6. Alternative Sources of Insulin-Producing Cells: Cell Replacement Therapy by Stem/Progenitor Cell-Derived Insulin-Producing Cells

Although islet transplantation is seen as a “cure” therapy for diabetes, this procedure is hampered by the limited number of donors for isolating islets. Many alternative approaches that can be applied to obtain insulin-secreting cells are being investigated intensively [58]. These include the following: (1) the production of surrogate cells by genetically modifying nonendocrine cells to secrete insulin in response to glucose challenge [59], (2) the transdifferentiation of nonendocrine stem/progenitor cells or mature cells to glucose-responsive adult tissues [60, 61], (3) the regulated differentiation of islet stem/progenitor cells to produce large numbers of mature, functional islets [62, 63], (4) the *in vitro* differentiation of stem cells to become insulin-secreting cells, and (5) the *in vitro* differentiation of induced pluripotent stem cells (iPSCs) derived from patients to form pancreatic *β*-like cells.

Stem cells can reproduce themselves (self-renew) and can differentiate into many cell types. These features make them an ideal focus for regenerative medicine. Besides, stem cells have strong immunosuppressive effects and can secrete many trophic factors that promote the regeneration of damaged tissues. Thus, stem cells have become an attractive alternative cell source to treat diabetes. There are many stem cell types available as a potential source for the generation of insulin-producing cells, including embryonic stem cells (ESCs), adult stem cells, and, most recently, the iPSCs.

Previous reports have demonstrated that ESCs can be induced to become insulin-secreting tissue with structures similar to pancreatic islets [64, 65], However, these cells are often unresponsive to glucose or produce lower levels of insulin compared with the endogenous *β* cells, which is insufficient to control normoglycemia in diabetic recipients in a mouse model [64]. ESCs have not yet been used therapeutically for treating diabetes mellitus in humans because the animal experiments have not progressed sufficiently to justify this approach; for example, the positive insulin staining of ESC-derived pancreatic-like tissue probably occurs by uptake of insulin from the culture medium [66], and the intermediate stages involved in the differentiation pathway are complicated and not fully understood [67]. In addition, the clinical applications of human ESCs are limited by ethical concerns, as well as the potential for teratoma formation.

In addition to the ESCs produced from embryos, mesenchymal stromal cells (MSCs) and iPSCs with fewer limitations or restrictions in ethical concerns in the research and clinical settings have become ideal cell types for regeneration therapies. Previous reports have demonstrated that MSCs can be differentiated into islet-like cell clusters, and these cell clusters can reverse the diabetic status after transplant into streptozotocin- (STZ-) induced diabetic recipients [68]. Moreover, MSCs themselves also have strong cytoprotective effects and immunosuppressive functions [69]. Several lines of evidence have demonstrated that cotransplantation of islets and MSCs produces superior outcomes to islet transplantation alone. In cotransplantation therapy, MSCs act as feeder cells that promote islet revascularization [70] and improve graft function [71]. Besides, MSCs also express immunoregulatory molecules, which might help to reestablish peripheral tolerance in diabetic recipients and to prevent allorejection [72].

Recently, iPSCs generated from reprogrammed somatic cells have become an exciting alternative source of cells with pluripotent characteristics similar to ESCs [73]. This concept has been tested further using human skin fibroblasts [74] or cells from skin biopsies from patients with T1D [75] to generate iPSC-derived islet-like clusters. These cells can secrete C-peptide and respond to high concentrations of glucose, suggesting that functional *β* cells might eventually be generated from iPSCs. In further studies, transplantation of iPSC-derived pancreatic beta-like cells reversed hyperglycemia in diabetic mouse models [76]. These data provide an initial proof of principle for the potential clinical application of iPSCs. Besides, if iPSCs could be generated from patients with diabetes, they would have the same genotype as the recipient, avoiding the problem of immune rejection. Thus, iPSCs show promise for patient-specific regenerative therapy. Unexpectedly and surprisingly, autologous iPSCs reprogrammed from fetal fibroblasts by viral or nonviral genetic approaches elicit T-cell-dependent immune reactions in genetically identical mice, resulting in their rejection [77]. This is likely because of the abnormal expression of antigens in the iPSCs, leading to a breakdown of peripheral tolerance. Besides, there could be other unknown epigenetic differences between iPSCs and ESCs [78].

## 7. Conclusion

T1D is among the most amenable diseases for treatment with cell replacement therapy. Clinical trials of islet transplantation are showing remarkable success since the Edmonton protocol was developed [79], and this glucocorticoid-free immunosuppressive protocol was replicated successfully [80]. However, the long-term success of this procedure is limited by the effects of allograft rejection and recurrent autoimmunity. Moreover, the scarcity of organ donors also frustrates the treatment of T1D. To overcome these problems, researchers have developed many strategies to modulate the detrimental immune responses and have also explored the alternative sources of insulin-secreting cells. Gene therapy offers a powerful tool to engineer islet grafts to become resistant to inflammation-induced apoptosis, as well as modifying islets to produce immunosuppressive molecules to attenuate T-cell response. The use of stem cells in the generation of renewable and functional *β* cells is now a promising reality. Moreover, based on current knowledge about genomic cell reprogramming, it should be possible to develop patient-specific, autologous cell replacement therapy by using iPSC-derived pancreatic *β*-like cells.

Although these proofs of concepts for potential preclinical applications show a big breakthrough in this field, some issues need to be considered: (1) the duration and expression levels of targeted genes in islets, (2) the use of viral vectors for direct gene therapy raises the possibility of insertional mutagenesis (retroviruses and lentiviruses) and host immunogenicity (adenoviruses), and (3) the efficiency of differentiation of insulin-secreting cells from stem cells.

In conclusion, further investigations are required to develop the most potent graft-specific immunoregulatory therapies and to generate safe and stable sources of insulin-secreting cells for clinical islet transplantation or cell replacement treatments.

## Figures and Tables

**Table 1:** Transgenic overexpression of regulatory genes in the islets and their effects on islet transplantation.

Promoter	Gene of interest	Animal strain	Diabetic incidence	Effect on islets	Effects on islet transplantation	Reference
Human insulin	IL-4	NOD	Decreased	Protect islets from autoimmune destruction	No significant protective effect	[21]
Rat insulin	TGF-*β*	NOD	Decreased	Small clusters of micro-islet	N, and no protective effect when use pancreata in an allogeneic transplantation model	[22, 23]
Glucagon	TGF-*β*	NOD	Decreased	Morphologically normal, no other phenotypes mentioned	N	[24]
Rat insulin	TNF-*α*	NOD	Decreased	Massive insulitis	N	[25]
Human insulin	SOCS1	B6	B6 is not a diabetes-prone mouse strain	Not mentioned	Expression of SOCS-1 in islets delays allografts rejection (B6 to Balb/c) but cannot circumvent destruction of the islets by the recurrence of the tissue-specific autoimmune process of spontaneous diabetes (B6 to diabetic NOD)	[26]
Human insulin	PD-L1	NOD	Decreased	Protect from autoimmune destruction	No significant protective effect	[27]
Glial fibrillary acidic protein	PD-L1	NOD	Increased	Enhance the severity of insulitis	N	[28]
Rat insulin	PD-L1	B6	Induces T-cell-mediated spontaneous diabetes in B6 mouse	Induce insulitis	Accelerate allograft rejection	[29]
Human insulin	Single chain anti-CTLA-4 Fv	NOD	Decreased	Protect islets from autoimmune destruction	Prolong islet grafts survival in diabetic NOD mice	[30]
Rat insulin	CTLA-4-Ig	B6	B6 is not a diabetes-prone mouse strain	Morphologically normal	N, and transplantation of CTLA4-Ig transgenic pancreata combine with transient systemic CD4 T cell depletion in recipients enhance allograft acceptance	[31]
Human insulin	Thioredoxin	NOD	Decreased	Do not attenuate the development of insulitis	N	[32]
Human insulin	Heme oxygenase 1	NOD	Decreased	Protect islets from autoimmune destruction Resistant to inflammatory cytokine-induced apoptosis	Prolong islet grafts survival in diabetic NOD mice	[33]
Human insulin	DcR3	NOD	Decreased	Protect islets from autoimmune destruction	Increase the successful rate of implantation and prolong islet grafts survival in diabetic NOD mice	[34]
Human insulin	D6	NOD	Decreased	Protect islets from autoimmune destruction	N	[35]

NOD: Nonobese diabetic mouse, a spontaneous autoimmune diabetes mouse strain; SOCS-1: suppressor of cytokine signaling-1; PD-L1: programmed death 1 ligand 1; CTLA-4: cytotoxic T lymphocyte antigen 4; DcR3: decoy receptor 3; D6: an inflammatory CC chemokine decoy receptor; N: not tested.

**Table 2:** Genetically engineered islets for transplantation therapy.

Vector type	Gene carried by vector	Effect on islet transplantation	Reference
Magnetic iron oxide nanoparticles	siRNA to caspase 3	Decrease cell apoptosis in recipients	[54]
Adenovirus	X-linked inhibitor of apoptosis protein (XIAP)	Increase successful rate of islet transplantation and reduce cell apoptosis in a syngeneic model	[55]
Gene gun transfection	CTLA-4-Ig	Prolong islet grafts' survival in an allogeneic model	[45]
Adenovirus/lentivirus	CTLA-4-Ig or TGF-*β*	Prolong islet grafts' survival in a xenogenetic model (rat to mouse)	[46]
Transfection by Lipofectin	Indoleamine 2, 3-dioxygenase (IDO)	Prolong islet grafts' survival in an allogeneic model	[56]
Adenovirus	TGF-*β*	Prolong islet grafts' survival in diabetic NOD mice	[51]
Adeno-associated virus	IL-10	Prolong islet grafts' survival in diabetic NOD mice	[52]
Adenovirus	IL-10	Combine with cyclosporin A, prolong islet grafts survival in an allogeneic model	[57]
Adenovirus	Bcl-2	Prolong islet grafts' survival and maintain functional islet mass in STZ-induced diabetic mice in a xenogenetic model (nonhuman primate to mouse)	[47]
Adenovirus	Manganese superoxide dismutase (MnSOD)	Prolong islet grafts' survival in STZ-induced diabetic NOD/SCID mice after challenge with diabetogenic splenocytes	[49]
Lentivirus	Thioredoxin	Prolong islet grafts' survival in diabetic NOD mice	[48]
Lentivirus	Galectin-9	Prolong islet grafts' survival in STZ-induced diabetic NOD/SCID mice after challenge with diabetogenic splenocytes	Chou et al., manuscript in preparation

## Abbreviations

T1D: Type 1 diabetes
NOD mouse: Nonobese diabetic mouse
PD-1: Programmed death-1
CTLA-4: Cytotoxic T-lymphocyte antigen-4
SOD: Superoxide dismutase
STZ: Streptozotocin
ESCs: Embryonic stem cells
MSCs: Mesenchymal stromal cells
iPSCs: Induced pluripotent stem cells.
